# Comments on “Economic Evaluation of Ultrasound-guided Central Venous Catheter Confirmation vs Chest Radiography in Critically Ill Patients: A Labor Cost Model”

**DOI:** 10.5811/westjem.2022.10.59187

**Published:** 2023-03-06

**Authors:** Samuel Austin, Quincy K. Tran, Ali Pourmand, Ann Matta, Daniel Haase

**Affiliations:** * University of Maryland School of Medicine, The R Adams Cowley Shock Trauma Center, Department of Surgical Critical Care, Baltimore, Maryland; † University of Maryland School of Medicine, Department of Emergency Medicine, Baltimore, Maryland; ‡ George Washington University School of Medicine and Health Sciences, Department of Emergency Medicine, Washington, DC

Dear Editors:

We would like to commend Dr. Ablordeppey and her colleagues for their recent publication in the *Western Journal of Emergency Medicine* evaluating the comparative labor cost of central venous catheter confirmation via point-of-care ultrasound (POCUS) vs traditional chest radiography (CXR).^1^ To our knowledge, this well-designed article was the first to analyze in detail the direct labor cost of using POCUS-guided confirmation for central lines vs traditional CXR confirmation. We were surprised when the authors reported that the POCUS-guided method was only $3.82 cheaper than the CXR method. With this small cost difference, low-volume hospitals that only perform a few hundred central lines per year may be less inclined to adopt this innovative method for reasons such as the cost of added ultrasound machines, formal appropriate ultrasound training of current staff, or medicolegal concerns. Perhaps this could be one of the barriers explaining the slow adoption of POCUS-guided central line confirmations among emergency physicians and intensivists.^2^

We noticed that the article provided rather conservative estimates of the 60-hour work week salary for the physicians who performed the procedure ($1.72 per minute for emergency physicians and $1.89 per minute for radiologists). The United States (US) Bureau of Labor Statistics reported the 2021 median hourly wage for emergency physicians and radiologists as $149.35 ($2.49 per minute) and $145.06 ($2.42 per minute), respectively.^3^,^4^ Furthermore, in the critical care resuscitation unit (CCRU) at the University of Maryland Medical Center, central lines were cannulated and confirmed mostly by our advanced practice practitioners (APP). This is a practice shared commonly with other institutions and settings.^5^–^7^ The US Bureau of Labor Statistics reported the 2021 median hourly wage for a nurse practitioner at $59.51 ($0.99 per minute) and for a physician assistant at $58.43 ($0.97 per minute).^8^,^9^ According to the calculations by Ablordeppey et al, the direct cost savings of POCUS-confirmation for central lines could be much greater for uncomplicated cases when they are performed by APPs ($10.56 or $10.45), as compared to the CXR method ($18.69). We acknowledge that a potential limitation to this suggestion is the lack of published data on the accuracy and feasibility of ultrasound-guided CVC confirmation that includes APPs as operators.

The application of a POCUS-guided method for central line placement would also offer significant savings in indirect costs. It has been established that it would take an average of 63.9 (± 57) minutes from the time of ordering the CXR to perform the CXR, compared with only 5.6 (± 2.5) minutes to perform a POCUS-guided technique to confirm central line placement.^10^ When factoring in the labor cost of a clinician waiting for CXR confirmation, this would represent another significant area of cost-saving. At the CCRU where critically ill patients are transferred for time-sensitive diseases,^11^ we receive hundreds of patients in extremis each year who need timely operative interventions. In certain instances, the CCRU team will insert central lines and perform POCUS-guided confirmation while the operating room is being prepped.

Although the coronavirus disease 2019 pandemic is slowing down, it’s still not over. Another potential example of indirect cost savings using a POCUS-guided central line confirmation strategy is minimizing the exposure of personnel and equipment to transmissible pathogens, subsequently reducing the need for personal protective equipment for staff and decontamination of the radiograph machines.

Therefore, we wholeheartedly agree with Dr. Ablordeppey and her colleagues that POCUS-guided central line confirmation is more efficient than the traditional CXR-guided method. The POCUS-guided method offers potential direct and indirect cost benefits when compared with the CXR method. We’d look forward to seeing more stakeholders move to adopt the POCUS method for central lines confirmation.
